# Safflower Yellow Pigment Alleviates Cerebral Ischemia‐Reperfusion Injury via Protein Nitration and Oxidative Modulation

**DOI:** 10.1002/brb3.70530

**Published:** 2025-05-08

**Authors:** Kun Wang, Xi Zhang, Yang Zhao, Lifeng Li, Ruijie Zhao

**Affiliations:** ^1^ Department of Neurology Xingtai Central Hospital Xingtai Hebei Province China; ^2^ Department of Neurology Xingtai People's Hospital Hebei Medical University Affiliated Hospital Xingtai Hebei Province China

**Keywords:** brain protein nitration and oxidative modification, cerebral ischemia‐reperfusion injury, neurological damage, oxidative stress, safflower yellow

## Abstract

**Objective:**

We seek to investigate the efficacy of safflower yellow pigment in mitigating cerebral ischemia‐reperfusion injury by examining its effects on protein nitration and oxidative modification.

**Methods:**

A total of 160 patients with acute ischemic stroke admitted to the department of neurology of Xingtai people's hospital were included in this study. This study was a retrospective study. Patients were divided into the control group (*n* = 80) and the observation group (*n* = 80) according to whether safflower yellow pigment was used. The control group received conventional treatment, and the observation group received safflower yellow pigment.

**Results:**

Baseline characteristics did not significantly differ between the two groups (*p* > 0.05). Serum nitrosine levels were lower in the observation group compared to the control group after 24 h and one week of treatment (*p* < 0.05). Similarly, serum carbonylated protein levels were lower in the observation group after 24 h and one week of treatment (*p* < 0.05). The observation group exhibited lower NIHSS and modified rankin scale (mRS) scores, reduced cerebral ischemic area. Furthermore, levels of malondialdehyde (MDA), C‐reactive protein (CRP), and tumor necrosis factor‐alpha (TNF‐α) were lower, while superoxide dismutase (SOD) activity was higher in the observation group compared to the control group after one week of treatment (*p* < 0.05).

**Conclusion:**

Safflower yellow pigment demonstrates significant neuroprotective effects in patients with cerebral ischemia‐reperfusion injury by reducing protein oxidation and nitration, improving neurological function, reducing cerebral ischemic area, and attenuating oxidative stress and inflammation.

## Graphical abstract

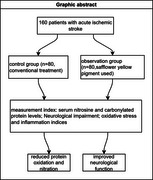

**Abbreviations**
CRP: C‐reactive protein
MDA: malondialdehyde
mRS: modified Rankin Scale
NO: nitric oxide
RNS: nitrogen oxides
ROS: reactive oxygen species
SOD: superoxide dismutase
TNF‐α: tumor necrosis factor‐alpha

## 1 Introduction

Saffron yellow pigment, derived from the stigma of saffron, has garnered significant interest in neuroprotection due to its potent antioxidant and anti‐inflammatory properties. These attributes render it promising for the treatment of various central nervous system disorders, particularly in mitigating cerebral ischemia‐reperfusion injury. Such injury, a common occurrence post‐stroke, involves intricate biochemical processes culminating in neuronal cell damage or demise, significantly impeding patients’ recovery and quality of life (Zhang et al. 2024; Fu and Liu 2021; Alsharabasy et al. 2022). The principal pathological mechanisms underlying cerebral ischemia‐reperfusion injury encompass oxidative stress and inflammatory cascades (Wasan et al. 2022). During this phase, an abundance of reactive oxygen species (ROS) and nitrogen oxides (RNS) is generated. These molecules attack cell membrane lipids, proteins, and nucleic acids, thereby inducing cellular dysfunction and demise. Among these, oxidative and nitroxidative alterations of proteins represent direct consequences of ROS and RNS actions, exacerbating cellular impairment. Leveraging the potent antioxidant attributes of saffron yellow pigment, it can directly scavenge ROS and RNS or augment the body's antioxidant enzyme activity, consequently curtailing cellular damage instigated by oxidative stress. Additionally, saffron yellow pigment safeguards nerve cells from harm by impeding the production and release of inflammatory mediators and mitigating inflammatory cell activation (Lipton 2022; Wang et al. 2024; Long et al. 2022). Despite the demonstrated neuroprotective efficacy of saffron yellow pigment in various studies, its clinical role and mechanism in assuaging cerebral ischemia‐reperfusion injury necessitate further exploration. This study aims to assess the impact of saffron yellow pigment on cerebral ischemia‐reperfusion injury in patients with acute ischemic stroke, with a specific focus on its regulatory effects on protein nitration and oxidative modification, and their correlation with clinical parameters such as neurological function enhancement and brain tissue damage reduction.

## 2 Materials and Methods

### 2.1 General Information of Patients

This was a retrospective study. A total of 160 patients with acute ischemic stroke admitted to the department of neurology of our hospital from december 2016 to debruary 2018 were included in this study. The sample size of 160 patients (80 per group) was determined based on the availability of complete medical records within the study period and alignment with a previous retrospective study investigating similar interventions (Lin et al. 2021). The patients, aged 28–73 years with a mean age of 54.35±4.26 years, comprised 89 males and 71 females. Patients were divided into the control group (*n* = 80) and the observation group (*n* = 80) according to whether safflower yellow pigment was used. The allocation to groups was based on historical medical records, and no prospective intervention was applied during the study period. All procedures were conducted in compliance with ethical norms and regulations.

### 2.2 Enrollment Criteria

1) Age 18 years or older, irrespective of gender.
2) Diagnosis of acute ischemic stroke based on clinical and imaging data.
3) Time from symptom onset to treatment initiation not exceeding 72 h.
4) Adequate verbal comprehension and expressive ability to facilitate accurate neurological scoring.
5) The pre‐onset mRS score was 0–1.

### 2.3 Exclusion Criteria

Patients meeting any of the following criteria were excluded from the study:

1) Presence of severe heart, liver, kidney, or blood system diseases, or other serious chronic illnesses that may impact study outcomes.
2) Concurrent diagnosis of other central nervous system disorders, such as brain tumors, myasthenia gravis, multiple sclerosis, etc.
3) Presence of severe cerebral edema, cerebral hemorrhage, etc.
4) Pregnancy or lactation.
5) History of known allergy to saffron yellow pigment.

### 2.4 Treatment Protocol

Patients in the observation group received safflower yellow pigment (specification: 50 mg/vial containing 42.5 mg hydroxysafflor yellow A [HSYA]; Jiangsu Caiwei Biotechnology Co., Ltd.) as an adjunct to conventional therapy. The intervention regimen followed the Chinese pharmacopoeia (2015 edition) guidelines (Committee 2015).

- Dosage: 100 mg/day (equivalent to two vials of 50 mg each), diluted in 250 mL of 0.9% sodium chloride injection.
- Administration: Intravenous drip at a rate ≤30 drops/minute, once daily.
- Treatment Duration: 14 consecutive days, constituting one full therapeutic cycle.

Patients in the control group received conventional treatment alone, including antiplatelet therapy, anticoagulation, and neuroprotective agents as per institutional protocols.

### 2.5 Brain Protein Nitration and Oxidative Modification Assays

Nitrotyrosine levels and carbonylated proteins in patient serum were assessed utilizing the ELISA technique. Blood samples were collected from patients and centrifuged to obtain serum, which was then stored at ‐80°C. Standard solutions of nitrotyrosine and carbonylatedproteins were prepared to establish a standard curve. Serum samples and standards were added to ELISA plates pre‐coated with antibodies to nitrotyrosine and carbonylated proteins, with duplicate wells to ensure accuracy. Samples were incubated at room temperature for 1 to 2 h to facilitate binding of nitrotyrosine and carbonylated proteins to solid‐phase antibodies, followed by washing to remove unbound material. Furthermore, enzyme‐labeled secondary antibody was added and incubated for 30 min to 1 h, followed by washing to remove unbound secondary antibody. Substrate TMB was added for color development, followed by incubation for a few minutes to 30 min and termination of the reaction.Finally, absorbance values were measured at 450 nm using an enzyme marker, and concentrations of nitrotyrosine and carbonylated proteins in serum were calculated from the standard curve to analyze changes before and after treatment.

### 2.6 Neurologic Function and Impairment Score Assessment

Following one week of treatment for acute ischemic stroke, patients underwent assessment of neurological impairment utilizing the NIHSS and modified rankin scale (mRS).The NIHSS provides a comprehensive evaluation of neurological function by assessing various parameters including consciousness, vision, motor function, sensation, speech, language, and attention. Each parameter is assigned a score, with higher scores indicating more severe neurological impairment. Specially trained medical personnel conducted detailed assessments to ensure accuracy of scoring. Conversely, the mRS evaluates the patient's ability to perform activities of daily living, ranging from zero (asymptomatic) to six (deceased), thereby reflecting the extent of functional limitation in daily life. Post‐treatment, NIHSS and mRS scores were recorded and compared with pre‐treatment scores. By calculating score changes and conducting statistical analysis, the treatment's effect on neurological function recovery could be quantified.

### 2.7 Magnetic Resonance Imaging Evaluation

After one week of acute ischemic stroke treatment, patients underwent MRI evaluation to assess cerebral ischemic area. Patients were ensured to have no contraindications to MRI examination and were instructed to remain still during scanning. MRI scanning involved the utilization of diffusion‐weighted imaging (DWI) to sensitively detect ischemic areas. DWI scanning was swiftly completed, typically within a few minutes.

### 2.8 Markers of Oxidative Stress and Inflammation Were Assessed

Venous blood samples (5–10 mL) were collected from patients and allowed to clot naturally for 30 min at room temperature. Subsequently, the samples were centrifuged at 3000 rpm for 10 min to separate the serum. MDA, SOD, CRP, and tumor necrosis factor‐alpha (TNF‐α) levels were determined using specific ELISA kits following the manufacturer's guidelines. This involved the preparation of standard curves and processing of samples. After incubation, washing, and addition of substrate for color development, absorbance values were measured using an enzyme marker to calculate the concentration of each indicator.

### 2.9 Statistical Analysis

This study used SPSS 20.0 statistical software; the measurement data was represented by “mean ± standard deviation” ($\bar{x}$±s), inter group comparisons are performed using one‐way ANOVA, and intergroup comparisons are performed using t‐test. The counting data is expressed as a percentage (%), and intergroup comparisons are made using χ^2^analysis. *p* < 0.05 represents a statistically significant difference.

## 3 Results

### 3.1 Comparison of Patients’ General Information

Statistical analysis revealed no significant differences in the general information between the two groups (*p* > 0.05). (Table 1)

**TABLE 1:** General statistics of patients.

Parameters	Control group (*n*=80)	Observation group (*n*=80)	t‐value/χ^2^ value	p‐value
Sex (M: F)	48:32	41:39	3.016	0.209
Age (years)	53.78 ± 5.27	54.85 ± 4.34	2.449	0.571
BMI (kg/m^2^)	22.16 ± 1.93	23.27 ± 2.12	5.617	0.384
Onset time (h)	14.62 ± 5.44	14.51 ± 6.56	1.445	0.224
NIHSS score	6.53 ± 0.64	6.61 ± 0.52	0.367	0.195
mRS score	3.16 ± 0.20	3.11 ± 0.16	1.275	0.292
Hypertension (%)	8 (10.00%)	10 (12.50%)	0.926	0.519
Diabetes (%)	7 (8.75%)	6 (7.50%)	1.264	0.445
Smoking (%)	23 (28.75%)	25 (31.25%)	0.746	0.206
Alcohol consumption (%)	26 (32.50%)	24 (30.00%)	1.1553	0.115

*Note*: Continuous variables are expressed as mean ± standard deviation; categorical variables as counts (percentage). 2. Independent samples *t*‐test used for age, BMI, onset time, NIHSS, and mRS scores; χ^2^ test used for sex, hypertension, diabetes, smoking, and alcohol consumption. 3. NIHSS: National Institutes of Health Stroke Scale; mRS: Modified Rankin Scale. 4. Baseline comparisons showed no significant differences between groups (*P* > 0.05 for all parameters).

### 3.2 Brain Protein Nitration Level Analysis

**FIGURE 1:**
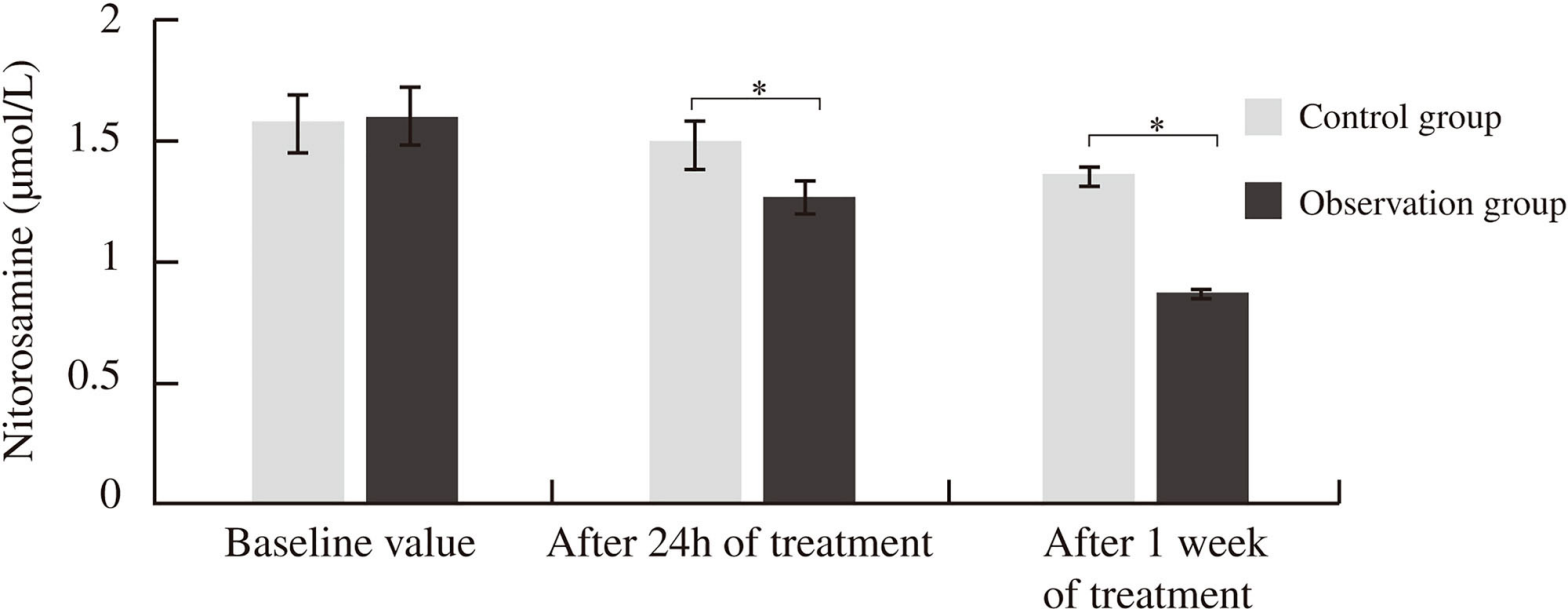
Comparison of serum nitrotyrosine levels between two groups at baseline and post‐treatment. ^*^ *p* < 0.05 observation vs Control.

Serumnitrotyrosine levels were comparable between the groups at baseline (*p* > 0.05). However, post‐treatment, nitrotyrosine levels were significantly lower in the observation group compared to the control group at both 24 h and one week (*p* < 0.05) (Figure 1, Table 2).

**TABLE 2:** The brain protein nitroxylated‐nitroxylated tyrosine levels.

Groups	Baseline value (µmol/L)	After 24 h of treatment (µmol/L)	After one week of treatment (µmol/L)
Control group (*n*=80)	1.58 ± 0.12	1.48 ± 0.10	1.35 ± 0.04
Observation group (*n*=80)	1.60 ± 0.13	1.27 ± 0.07	0.86 ± 0.02
t‐value	0.836	11.455	16.581
*p*‐value	0.216	0.001	0.000

*Note*: Values expressed as mean ± standard deviation (µmol/L). Between‐group comparisons analyzed using independent samples *t*‐test. *P* < 0.05 considered statistically significant.

### 3.3 Analysis of Oxidative Modification of Brain Proteins

Serumcarbonylated protein levels were measured using ELISA, revealing no significant difference in baseline levels between the two patient groups (*p* > 0.05). However, after 24 h and one week of treatment, the observation group exhibited lower serum carbonylated protein levels compared to the control group (*p* < 0.05). (Table 3)

**TABLE 3:** Analysis of brain protein oxidative modification‐carbonylation protein levels.

Groups	Baseline value (µmol/L)	After 24 h of treatment (µmol/L)	After one week of treatment (µmol/L)
Control group (*n*=80)	2.48 ± 0.32	2.41 ± 0.23	2.26 ± 0.19
Observation group (*n*=80)	2.50 ± 0.34	2.11 ± 0.18	1.44 ± 0.10
t‐value	0.836	11.455	16.581
*p*‐value	0.216	0.002	0.000

*Note*: Values expressed as mean ± standard deviation (µmol/L). Independent samples *t*‐test used for between‐group comparisons. *P* < 0.05 considered statistically significant.

### 3.4 Neurologic Function Andimpairment Score

Following one week of treatment, neurological impairment was assessed using the NIHSS and mRS. The observation group demonstrated lower NIHSS and mRS scores compared to the control group (*p* < 0.05), as depicted in Table 4.

**TABLE 4:** NIHSS and mRS scores.

Groups	NIHSS	mRS
Control group (*n*=80)	4.56 ± 0.83	2.01 ± 0.15
Observation group (*n*=80)	3.52 ± 0.62	1.86 ± 0.10
t‐value	10.525	17.205
*p*‐value	0.001	0.000

*Note*: NIHSS: National Institutes of Health Stroke Scale (range: 0–42, higher scores indicate greater severity). mRS: Modified Rankin Scale (range: 0–6, higher scores indicate worse functional outcomes). Independent samples *t*‐test used for comparisons; *P* < 0.05 considered significant.

### 3.5 Magnetic Resonance Imaging Analysis

After one week of treatment, there was a significant difference in the MRI evaluation of cerebral ischemic area between the observation group and the control group. Compared with the control group, the observation group showed a decrease in cerebral ischemic area (*p* < 0.05). (Table 5)

**TABLE 5:** Analysis of brain imaging results.

Groups	Cerebral ischemic area (cm^2^)
Control group (*n*=80)	4.25 ± 0.22
Observation group (*n*=80)	3.68 ± 0.15
t‐value	15.186
*p*‐value	0.000

*Note*: Cerebral ischemic area measured in cm^2^ and expressed as mean ± standard deviation. Independent samples *t*‐test used for between‐group comparison. *P* < 0.001 indicates statistically significant reduction in the observation group.

### 3.6 Analysis of Oxidative Stress and Inflammatory Markers

Comparison of oxidative stress and inflammatory markers between the two groups after one week of treatment revealed significant differences. Levels of MDA, CRP, and TNF‐α were lower in the observation group compared to the control group (*p* < 0.05). Additionally, SOD activity was higher in the observation group (*p* < 0.05). (Table 6)

**TABLE 6:** Markers of oxidative stress and inflammation.

Groups	MDA (µmol/L)	SOD (U/ml)	CRP (mg/L)	TNF‐α (pg/ml)
Control group (n=80)	2.86 ± 0.13	126.25 ± 10.44	2.21 ± 0.14	37.69 ± 4.03
Observation group (n=80)	2.05 ± 0.08	196.34 ± 15.72	1.45 ± 0.10	24.63 ± 3.26
t‐value	10.811	14.295	16.334	12.055
P‐value	0.002	0.001	0.000	0.001

Abbreviations: MDA: Malondialdehyde; SOD: Superoxide Dismutase; CRP: C‐reactive protein; TNF‐α: Tumor Necrosis Factor‐alpha. Values expressed as mean ± standard deviation. Independent samples *t*‐test used for comparisons; *P* < 0.05 considered significant.

## 4 Discussion

Acute ischemic stroke, a prevalent cerebrovascular disorder, occurs when blood flow to a specific brain region is abruptly obstructed, depriving corresponding brain tissues of adequate oxygen and nutrients, thereby instigating brain cell damage or demise. Notably, nitration and oxidative modification of brain proteins play pivotal roles in this process, constituting significant molecular mechanisms underlying cerebral ischemia‐reperfusion injury (Chang et al. 2022, Shen et al. 2024). These mechanisms are well‐documented in preclinical models, where protein nitration exacerbates mitochondrial dysfunction and neuronal apoptosis (Chen et al. 2011). Post‐cerebral ischemia, timely restoration of blood flow (reperfusion) theoretically offers the potential to salvage brain cells within the ischemic region. However, reperfusion itself may precipitate additional damage, termed ischemia‐reperfusion injury (Zhang et al. 2022). During reperfusion, a surge of oxygen free radicals surpasses the brain tissue's scavenging capacity, inducing oxidative stress. These radicals assail lipids, proteins, and DNA within brain cells, eliciting cellular dysfunction or demise. Notably, nitrosylation of brain proteins, particularly tyrosine residues, by nitric oxide (NO) and its derivatives to form nitrotyrosine (3‐nitrotyrosine), represents a crucial facet of oxidative stress. This modification disrupts protein structure and function, potentially perturbing cell signaling, enzyme activity, and cytoskeletal integrity, thereby impacting cell survival (Su et al. 2022; Chen et al. 2024). Recent studies emphasize nitrotyrosine as a prognostic biomarker in ischemic stroke, correlating with infarct volume and neurological outcomes (Chen et al. 2020).

Concurrently, oxidative modification of brain proteins is also integral to ischemia‐reperfusion injury, precipitating alterations in protein structure and function, thereby impeding normal physiological brain cell activities. Pharmacologically, saffron yellow pigment exhibits diverse biological activities encompassing antioxidant, anti‐inflammatory, antidepressant, and neuroprotective effects (You et al. 2023). Its antioxidant properties are attributed to crocin and crocetin, bioactive constituents shown to neutralize ROS and upregulate endogenous antioxidants like glutathione in rodent stroke models (Wu et al. 2023). Furthermore, its anti‐inflammatory effects involve suppressing inflammatory mediator production and modulating inflammatory signaling pathways. Within the nervous system, saffron yellow pigment exerts notable neuroprotective effects (Chen et al. 2023; Shan and Ge 2022), attenuating cerebral ischemia‐reperfusion injury, conceivably via reduction of oxidative stress and inflammatory responses within brain tissue.

The objective of this study was to assess the impact of saffron yellow pigment on cerebral ischemia‐reperfusion injury in patients with acute ischemic stroke, with a particular focus on its influence on protein nitration and oxidative modification. Through a comprehensive analysis of 160 patients, we observed significant improvements in several crucial biomarkers in the saffron yellow pigment‐treated group compared to the control group, thereby furnishing compelling evidence for the potential therapeutic role of saffron yellow pigment as an adjunctive therapy for ischemic stroke. Notably, serum levels of nitrotyrosine and carbonylated proteins were markedly lower in the saffron yellow pigment‐treated observation group, indicating its potential in mitigating protein oxidation and nitroxylation modifications. These results resonate with findings from a recent clinical trial where crocin supplementation reduced oxidative protein modifications in diabetic patients with cardiovascular comorbidities (Karimi‐Nazari et al. 2019). These findings not only align with the well‐established antioxidant and anti‐inflammatory properties of saffron yellow pigment but also furnish empirical support for its neuroprotective application (Zhang and Gong 2024; Huang et al. 2024).The pivotal role of oxidative stress in ischemia‐reperfusion injury cannot be overstated, as it entails the generation of copious reactive oxygen species (ROS) capable of assaulting proteins, lipids, and nucleic acids, thereby exacerbating cellular damage and death (Xiao et al. 2023). Oxidative modifications of proteins, such as carbonylation, perturb protein structure and function, thereby disrupting essential cellular processes. Similarly, nitroxylation modifications can impede protein function and activity, exacerbating cellular damage. This aligns with mechanistic studies demonstrating crocetin's ability to preserve protein integrity by inhibiting ROS‐mediated carbonylation in neuronal cells (Chang et al. 1996). By curtailing the incidence of these modifications, saffron yellow pigment may uphold normal protein function and cellular homeostasis. The antioxidant prowess of saffron yellow pigment may stem from its unique chemical structure, which may facilitate direct scavenging of free radicals or augmentation of the body's endogenous antioxidant defense system. Additionally, its anti‐inflammatory effect may entail inhibition of inflammatory mediator production or suppression of inflammatory cell activation, thereby conferring protection during ischemia‐reperfusion events.

The NIHSS and mRS scoring systems, pivotal for evaluating neurological deficits and limitations in daily living tasks among stroke patients (Wang et al. 2023; Lu et al. 2022), exhibited notable improvement in our study, further affirming the neuroprotective efficacy of saffron yellow pigment.Reductions in NIHSS scores reflect substantial enhancements across various domains including language, consciousness, visual and motor function, while improvements in mRS scores indicate enhanced self‐care abilities in daily life for patients. These findings are consistent with a recent study of herbal interventions in stroke rehabilitation, where saffron derivatives were associated with improved functional outcomes (Wen et al. 2020). These score alterations directly correlate with patients’ quality of life and rehabilitation outlook, underscoring the potential therapeutic utility of saffron yellow pigment in fostering neurological recuperation. Consistent with clinical scoring system outcomes, MRI imaging results also underscore the beneficial impact of saffron yellow pigment. The observed reduction in cerebral ischemic area post‐treatment in the observation group suggests saffron yellow pigment's potential in mitigating the spread of ischemic injury. This parallels preclinical evidence where crocin reduced infarct volume in middle cerebral artery occlusion models by enhancing cerebral perfusion and attenuating blood‐brain barrier disruption (Vakili et al. 2014). These physiological and biochemical shifts suggest that saffron yellow pigment may confer protective effects by facilitating blood flow restoration and diminishing cellular damage. These findings closely align with the mechanisms by which saffron yellow pigment mitigates oxidative stress and inflammatory responses (Davey and Mattson 2022; Wang et al. 2023). Oxidative stress and inflammation represent pivotal pathological processes in ischemia‐reperfusion injury, and by tempering these processes, saffron yellow pigment may attenuate cellular damage, fostering the survival and functional recovery of nerve cells.

MDA serves as a direct marker of lipid peroxidation within cell membranes, commonly utilized to gauge levels of oxidative stress within the body (Zhang et al. 2022). SOD, on the other hand, is a crucial antioxidant enzyme that scavenges harmful superoxide radicals, thereby safeguarding cells against oxidative harm (Yasui and Baba 2006). The observed capability of saffron yellow pigment to diminish MDA levels while enhancing SOD activity suggests its efficacy in mitigating cellular oxidative stress while fortifying the body's antioxidant defenses. Furthermore, the anti‐inflammatory properties of saffron yellow pigment are underscored by reductions in CRP and TNF‐α levels. CRP, an acute phase protein, exhibits heightened plasma concentration during inflammatory responses (Black et al. 2004), while TNF‐α functions as a pivotal inflammatory cytokine governing inflammatory reactions across various cell types. The observed reduction in TNF‐α aligns with prior preclinical studies demonstrating saffron's ability to suppress NF‐κB signaling, a key regulator of TNF‐α synthesis (Wang et al. 2020; Banskota et al. 2021). Similarly, the decline in CRP levels corroborates clinical evidence linking saffron derivatives to attenuated systemic inflammation in cardiovascular diseases (Su et al. 2021; Razavi et al. 2017). While these findings suggest saffron yellow pigment modulates inflammatory pathways, further investigation into its effects on broader cytokine networks (e.g., IL‐6, IL‐1β) and intracellular signaling cascades is warranted to fully elucidate its anti‐inflammatory mechanism. These biochemical alterations align harmoniously with the established pharmacological profile of saffron yellow pigment, which not only serves as a potent scavenger of free radicals to mitigate oxidative stress but also exerts anti‐inflammatory effects through modulation of inflammation‐related mediators (Hou et al. 2023; Saini et al. 2023).

To conclude, the present study underscores the potential of saffron yellow pigment in ameliorating cerebral ischemia‐reperfusion injury through its ability to mitigate oxidative stress and inflammatory responses, diminish protein oxidation and nitroxylation modifications, and enhance cerebral hemodynamics. These findings advocate for the consideration of saffron yellow pigment as an adjunctive therapy for acute ischemic stroke. However, we acknowledge the limitations of this study, including its single‐center design and relatively small sample size (*n* = 160). Future research should prioritize multi‐center studies with larger cohorts to validate our findings and improve their generalizability. Additionally, further investigations into the specific molecular mechanisms underlying the effects of saffron yellow pigment on protein nitration and oxidative modification are warranted to provide deeper insights into its therapeutic potential.

## Author Contributions

**Kun Wang**: conceptualization, writing – review and editing, writing – original draft. **Xi Zhang**: investigation, methodology. **Yang Zhao**: funding acquisition, validation. **Lifeng Li**: visualization, software. **Ruijie Zhao**: formal analysis, project administration, writing – review and editing.

## Ethics Statement

The current study was conducted in accordance with the Helsinki Declaration of the World Medical Association and approved by the Ethics Committee of Xingtai People's Hospital. Informed consent was obtained from all the study subjects before enrollment.

## Consent

The authors have nothing to report.

## Conflicts of Interest

The authors declare no conflicts of interest.

### Peer Review

The peer review history for this article is available at https://publons.com/publon/10.1002/brb3.70530.

## Data Availability

The datasets generated and analyzed during the current study are available from the corresponding author on reasonable request.
